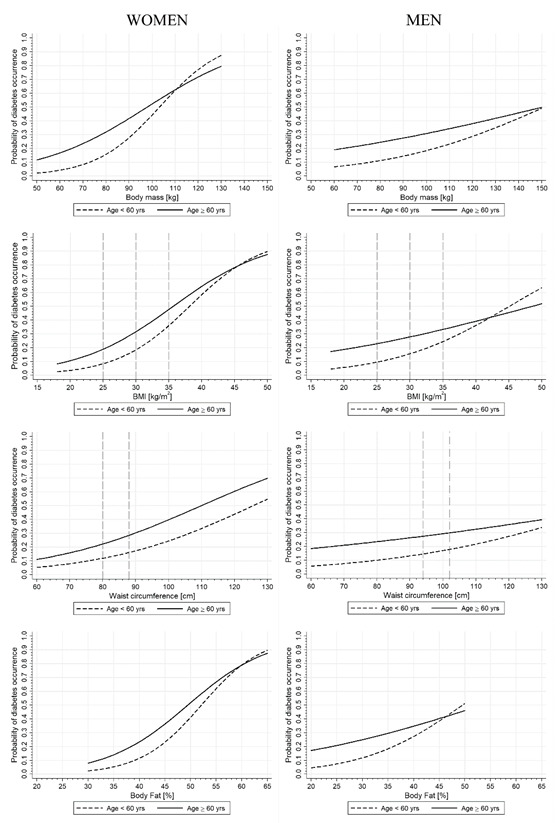# Correction: Bednarek et al. The Prevalence of Diabetes among Hypertensive Polish in Relation to Sex-Difference in Body Mass Index, Waist Circumference, Body Fat Percentage and Age. *Int. J. Environ. Res. Public Health* 2022, *19*, 9458

**DOI:** 10.3390/ijerph20105834

**Published:** 2023-05-16

**Authors:** Anna Maria Bednarek, Aleksander Jerzy Owczarek, Anna Chudek, Agnieszka Almgren-Rachtan, Katarzyna Wieczorowska-Tobis, Magdalena Olszanecka-Glinianowicz, Jerzy Chudek

**Affiliations:** 1 First Department of Cardiology, Medical University of Silesia in Katowice, 40-635 Katowice, Poland; 2 Health Promotion and Obesity Management Unit, Department of Pathophysiology, Faculty of Medical Sciences in Katowice, Medical University of Silesia in Katowice, 40-752 Katowice, Poland; 3 Department of Pharmacovigilance, Europharma Rachtan Co., Ltd., 40-061 Katowice, Poland; 4 Laboratory for Geriatric Medicine, Department of Palliative Medicine, University of Medical Sciences, 60-355 Poznan, Poland; 5 Department of Internal Medicine and Oncological Chemotherapy, Faculty of Medical Sciences in Katowice, Medical University of Silesia in Katowice, 40-029 Katowice, Poland

## Figure Legend

In the original publication [1], there were mistakes in Figure 3. Figure column titles of ‘women’ and ‘men’ were missing. The X axis of the Body Mass graph for men was also mislabeled as “Waist circumference (cm)”, and the x axis bounds were too short. The Women’s Waist circumference graph was also a duplicate of the Men’s Waist circumference graph. The corrected Figure 3 appears below. The authors have also swapped the order of the Body mass (kg) and BMI (kg/m^2^) rows to match with the order presented in previous tables. The authors state that the scientific conclusions are unaffected. This correction was approved by the Academic Editor. The original publication has also been updated.